# Comparison Between QT and Corrected QT Interval Assessment by an Apple Watch With the AccurBeat Platform and by a 12‑Lead Electrocardiogram With Manual Annotation: Prospective Observational Study

**DOI:** 10.2196/41241

**Published:** 2022-09-28

**Authors:** Sara Chokshi, Gulzhan Tologonova, Rose Calixte, Vandana Yadav, Naveed Razvi, Jason Lazar, Stan Kachnowski

**Affiliations:** 1 Healthcare Innovation and Technology Lab New York, NY United States; 2 Division of Cardiovascular Medicine State University of New York Downstate Medical Center New York, NY United States; 3 Department of Epidemiology and Biostatistics State University of New York Downstate Health Sciences University New York, NY United States; 4 Department of Cardiology Ipswich Hospital Ipswich United Kingdom; 5 Columbia Business School Columbia University New York, NY United States; 6 Indian Institute of Technology Delhi Delhi India

**Keywords:** artificial intelligence ECG, AI ECG, AI wearables, big data, cardiovascular medicine, digital health, machine learning

## Abstract

**Background:**

Abnormal prolongation or shortening of the QT interval is associated with increased risk for ventricular arrhythmias and sudden cardiac death. For continuous monitoring, widespread use, and prevention of cardiac events, advanced wearable technologies are emerging as promising surrogates for conventional 12‑lead electrocardiogram (ECG) QT interval assessment. Previous studies have shown a good agreement between QT and corrected QT (QTc) intervals measured on a smartwatch ECG and a 12-lead ECG, but the clinical accuracy of computerized algorithms for QT and QTc interval measurement from smartwatch ECGs is unclear.

**Objective:**

The prospective observational study compared the smartwatch-recorded QT and QTc assessed using AccurKardia’s AccurBeat platform with the conventional 12‑lead ECG annotated manually by a cardiologist.

**Methods:**

ECGs were collected from healthy participants (without any known cardiovascular disease) aged >22 years. Two consecutive 30-second ECG readings followed by (within 15 minutes) a 10-second standard 12-lead ECG were recorded for each participant. Characteristics of the participants were compared by sex using a 2-sample *t* test and Wilcoxon rank sum test. Statistical comparisons of heart rate (HR), QT interval, and QTc interval between the platform and the 12-lead ECG, ECG lead I, and ECG lead II were done using the Wilcoxon sign rank test. Linear regression was used to predict QTc and QT intervals from the ECG based on the platform’s QTc/QT intervals with adjustment for age, sex, and difference in HR measurement. The Bland-Altman method was used to check agreement between various QT and QTc interval measurements.

**Results:**

A total of 50 participants (32 female, mean age 46 years, SD 1 year) were included in the study. The result of the regression model using the platform measurements to predict the 12-lead ECG measurements indicated that, in univariate analysis, QT/QTc intervals from the platform significantly predicted QT/QTc intervals from the 12-lead ECG, ECG lead I, and ECG lead II, and this remained significant after adjustment for sex, age, and change in HR. The Bland-Altman plot results found that 96% of the average QTc interval measurements between the platform and QTc intervals from the 12-lead ECG were within the 95% confidence limit of the average difference between the two measurements, with a mean difference of –10.5 (95% limits of agreement –71.43, 50.43). A total of 94% of the average QT interval measurements between the platform and the 12-lead ECG were within the 95% CI of the average difference between the two measurements, with a mean difference of –6.3 (95% limits of agreement –54.54, 41.94).

**Conclusions:**

QT and QTc intervals obtained by a smartwatch coupled with the platform’s assessment were comparable to those from a 12-lead ECG. Accordingly, with further refinements, remote monitoring using this technology holds promise for the identification of QT interval prolongation.

## Introduction

Cardiovascular disease is highly prevalent and poses significant morbidity and mortality, accounting for approximately 1 in every 4 deaths in the United States alone [1,2]. Remote monitoring of heart health to detect early signs of deterioration and thus opportunities for intervention prior to situations requiring inpatient care is an area that would benefit from a cost-saving outcome-improving innovation.

The 12-lead electrocardiogram (ECG) has long been a standard component of evaluation for patients suspected of or confirmed to have cardiovascular disease. However, use of the 12-lead ECG is restricted to medical facilities, as qualified physicians are needed to interpret the results [3-5]. In low- and middle-income countries, where over 75% of deaths are related to cardiovascular disease and where there is limited access to ECG equipment and cardiologists, many patients with heart disease need regular ECG checks and reviews, both to check for disease progression and for surveillance of drug therapies [6]. This demand can place an insurmountable burden on the available pool of specialists, including in areas with a high concentration of populations susceptible to heart disease (eg, South Asian people), particularly in the COVID-19 pandemic era [7].

The QT interval is an important parameter derived from the 12-lead ECG that represents the time for ventricular depolarization. A key data point of interest in the ECG is the QT interval [8-10]. While most commonly corrected for heart rate (HR), abnormal prolongation or shortening of the QT interval, whether congenital [11,12] or acquired, is associated with increased risk for ventricular arrhythmias and even sudden cardiac death [13].

The QT interval is also an important parameter to follow in patients treated with cardiac medications. Advanced wearable technologies provide new opportunities for the diagnosis and management of cardiovascular diseases and their risk factors in the convenience of patients’ homes and other nonclinical settings [14]. The latest generation of smartwatches and smartphones are increasingly popular tools for health monitoring and care delivery, capable of collecting key vital signs such as HR, blood pressure, and even ECG data [15]. The Apple Watch, which recently received approval for atrial fibrillation detection from the Food and Drug Administration, can perform an ECG using a single peripheral lead (lead I)—obtained through a circuit between the detector on the back of the watch and the digital crown. While the Apple Watch single-lead ECG can detect atrial fibrillation, the feasibility and diagnostic accuracy for QT interval measurement is less established [10,16]. While previous studies have already shown a good agreement between QT and corrected QT (QTc) intervals measured on a smartwatch ECG and a 12-lead ECG [17], the use of computerized algorithms for QT and QTc interval measurement from smartwatch ECGs lacks a similar level of evidence [18]. This is a key step in fulfilling the promise of using wearable technologies to facilitate the diagnosis and management of cardiovascular health [19].

To build upon the promise of this new technology, a cardiology-focused digital health company (AccurKardia) has developed a device agnostic platform (AccurBeat) for the analysis of Apple Watch (version 4 or higher)–generated ECGs that leverages an engine built on computational and artificial intelligence (AI) techniques to perform automated analysis of ECGs and support the early detection and diagnosis of arrhythmias. The objective of this study is to compare smartwatch-recorded QT and QTc intervals assessed using the platform’s algorithm with the conventional gold standard procedure that uses a 12‑lead ECG annotated manually by an expert cardiologist.

## Methods

### Study Design

This study is a single-site observational study to compare QT and QTc intervals assessed using smartwatch-generated data coupled with the platform’s algorithm and QT and QTc intervals measured using a 12‑lead ECG with manual annotation in healthy individuals. The study was performed in the Noninvasive Cardiology Unit at the State University of New York (SUNY) Downstate Medical Center in Brooklyn, New York, a large urban medical center.

### Ethics Approval

The study was deemed as human participants research and was approved by the Biomedical Research Alliance of New York Institutional Review Board and the Institutional Review Board at SUNY Downstate Medical Center (IRB 21-02-474), and all participants provided informed consent that was delivered and documented by the study coordinator prior to data collection. All participant data was collected anonymously. Aside from the Apple Watch–generated readings, data were recorded and stored securely on SUNY Downstate Medical Center servers in password-protected spreadsheets. Apple Watch–generated data, once captured, was automatically sent through an API call to a cloud-based analytics engine (“AccurAI”), which annotated the ECG and provide a computerized interpretation of the rhythm that was identified. The output of these analytics was accessible through a secure web-based clinician portal for the AccurBeat device.

### Study Population

Healthy adult participants without known or suspected heart disease were recruited from outpatient primary care and cardiology clinics between January 6 and 19, 2022. This was a convenience sample from the SUNY Downstate cardiology clinic and internal medicine practice. Patients were screened by the study coordinator daily prior to their visit and, if qualified, were informed of the study. In total, 54 patients were screened, and 50 patients provided informed consent. The inclusion criteria were selected based on both patient self-report and electronic medical records. The exclusion criteria included any recent illness within 4 weeks and taking any medication irrespective of an indication that is known to prolong the QT interval.

### Study Procedure

#### Data Collection

Each participant was informed about the study procedures, and written consent was collected. Each participant was then asked to sit down while a highly experienced study coordinator placed the study-dedicated Apple Watch (version 7) on their left wrist and facilitated two consecutive 30-second ECG readings. Within 15 minutes following the Apple Watch readings, the study coordinator had participants lie flat and proceeded to place electrodes on the participants to perform a 10-second standard 12-lead ECG reading using the GE MAC 5500HD ECG machine with a paper speed of 25 mm per second. All participants were compensated (US $75) via gift card following their study visit.

#### Data Storage and Analysis

Apple Watch ECG data was automatically uploaded to Apple HealthKit, Apple’s central repository for health and fitness data on the iPhone and was confirmed immediately following collection by the study coordinator via the platform’s smartphone app. The 12-lead ECG readings were printed at the time of reading and labeled with the data and time of reading for identification purposes. All Apple Watch ECG data were assessed using the platform. The corresponding 12-lead ECG was assessed and manually annotated by an expert cardiologist.

#### Annotation Procedures

The ECGs were recorded on paper tracings. They were digitalized and then imported in ImageJ (free online software provided by the National Institutes of Health). The calibrations were performed for 0.4 seconds, and the followings measures were made for each beat in each lead: in QQ interval and in respiratory rate interval.

All ECGs had a placing that was technically adequate for analysis. In the case of a flattened T wave, the lead was excluded for analysis from the QT interval of the 12 leads. All analyzable complexes were in lead I and lead II. Bazzett’s [20] formula was used to correct for HR in all determinations.

#### Solution Development and Evaluation

The AccurBeat (version 1.0) platform includes a native iOS app (used to view the annotated ECG and computerized interpretation of rhythm classification), a clinician web portal (for the review and approval of reports prior to release to patients), a cloud-based application programming interface to access the analytics engine, and the analytics engine itself that annotates the ECG and provides a computerized interpretation of rhythm classification. The analytics engine is based on proprietary methods that leverage a combination of signal processing, image analysis, and AI-based techniques to annotate ECGs and diagnose arrhythmias. The data is normalized, and features are extracted using various signal processing techniques. Once this initial processing is complete, a hybrid architecture combining image analysis with evolutionary computing–based AI is invoked for beat classification, complex feature extraction, and rhythm detection. Following this, an inference engine with established clinical guidelines is used to obtain a diagnosis. Since this study only focused on HR, QT interval, and QTc interval measurements, the output of the inference engine was not applicable to the results of this study. The algorithm was previously tested according to the AAMI ANSI EC57:2012 standard with both publicly available and proprietary databases.

#### Statistical Analysis

Quantitative variables (age, HR, QT interval, and QTc interval) are summarized as means (SD) or median (IQR), and categorical variables are reported as frequencies (percentages). We compared characteristics of the participants by sex using a 2-sample *t* test and Wilcoxon rank sum test. Statistical comparisons of HR, QT interval, and QTc interval between the platform and 12-lead ECG, ECG lead I, and ECG lead II were done using the Wilcoxon sign rank test. We used linear regression to predict the QTc and QT intervals from the ECG based on the platform’s QTc/QT intervals, with adjustment for age, sex, and the difference in HR measurement. We checked for multicollinearity using variance inflation factor (VIF).

Agreement between QT and QTc interval measurements (taken on 12-lead ECGs and annotated manually and taken on smartwatches and assessed by the platform) was assessed using the Bland-Altman method [21,22]. The mean of the difference (bias) in QT and QTc intervals between the two methods was calculated, along with the 95% lower and upper limits of agreement (LoA). Agreement between the measures was also numerically assessed by estimating the agreement intraclass correlation coefficient, with its 95% CI [23]. Statistical significance was set at .05. All analyses were done in R 4.0.3 (R Foundation for Statistical Computing) and RStudio 1.2.5019 (RStudio, PBC).

## Results

In Table 1, we summarized the characteristics of the study participants and compared them by sex. Of all 50 participants, 32 (64%) of the study participants identified as female. They had a mean age of 46.18 (11.89) years. There was no sex difference in mean age, mean HR measurements from all devices, or mean QT interval measurements from all devices. However, QTc interval measurements from all devices were significantly higher for female patients compared to male patients.

In Table 2, we summarized HR, QT intervals, and QTc intervals between the platform and 12-lead ECG, ECG lead I, and ECG lead II. Results of the Wilcoxon sign rank test indicated that all measurements from the AccurBeat device were significantly higher than those from the 12-lead ECG.

The correlations, with 95% CIs, between the platform measurements and 12-lead ECG measurements are featured in Table 3. The result indicated that the correlations between measurements across devices were all significantly different from 0. However, the strengths of association range from low to strong positive associations. In the sensitivity analysis, the results remained consistent.

The result of the regression model using the platform measurements to predict the 12-lead ECG measurements (Table 4) indicate that, in univariate analysis, QT/QTc intervals from the platform significantly predicted QT/QTc intervals from the 12-lead ECG, ECG lead I, and ECG lead II. The significant association between QT/QTc intervals from the platform and QT/QTc intervals from the 12-lead ECG remained significant after adjustment for sex, age, and change in HR. In the multivariable model, for each unit increase in the platform QTc interval, the QTc interval from the 12-lead ECG was expected to increase by 0.31 (adjusted *R*^2^=0.38). Similarly, the QTc interval from ECG lead I and ECG lead II were expected to increase significantly by 0.30 (adjusted *R*^2^=0.25 and 0.32 for lead I and lead II, respectively). A 33-point increase in the platform QTc interval would correspond to approximately a 10-point increase in the QTc interval from the 12-lead ECG, adjusting for age, sex, and change in HR. In the multivariable model, for each unit increase in the platform QT interval, the QT interval from the 12-lead ECG was expected to increase by 0.53 (adjusted *R*^2^=0.47). Similarly, the QT interval from ECG lead I and ECG lead II were expected to increase significantly by 0.43 and 0.48 (adjusted *R*^2^=0.39 and 0.29), respectively. A 19-point increase in the platform QT interval (24 for lead I and 21 for lead II) would correspond to an approximately 10-point increase in the QT interval from the 12-lead ECG, adjusting for age, sex, and change in HR. The VIF for all six models was less than 2, indicating no multicollinearity.

**Table 1:** Descriptive summary of age, HR, QT interval, and QTc interval by sex.

	Female (n=32)	Male (n=18)	*P* value^a^
Age (years), mean (SD)	47.44 (10.99)	43.94 (13.38)	.35
Platform HR^b^ (bpm), mean (SD)	78.67 (9.92)	74.50 (11.79)	.21
Platform QT interval (ms), median (IQR)	401 (371.8-424)	374 (369.1-392.4)	.10
Platform QTc^c^ interval (ms), median (IQR)	444.5 (433.4-465.2)	423.8 (410.2-434.2)	<.001
12-lead HR (bpm), mean (SD)	75.22 (9.99)	73.00 (11.34)	.49
12-lead QT interval (ms), median (IQR)	382 (363.5-410.5)	375 (360-388)	.29
12-lead QTc interval (ms), median (IQR)	431.5 (412-441)	410.5 (40.32-415.8)	.006
Lead I HR (bpm), mean (SD)	77.42 (11.62)	72.17 (12.72)	.16
Lead I QT interval (ms), median (IQR)	366.5 (350.8-393.2)	363.5 (349.5-381)	.56
Lead I QTc interval (ms), median (IQR)	418 (406.5-435)	397 (373.5-718.8)	.02
Lead II HR (bpm), mean (SD)	76.68 (10.34)	72.06 (14.38)	.24
Lead II QT interval (ms), median (IQR)	371 (356-396.2)	362.5 (353.2-380.2)	.18
Lead II QTc interval (ms), median (IQR)	430 (410-436.5)	388 (366.5-410.2)	.003

^a^Statistical comparison between measurements from the platform and the 12-lead electrocardiogram were done using a 2-sample *t* test and Wilcoxon rank sum test. A *P* value <.05 was considered significant.

^b^HR: heart rate.

^c^QTc: corrected QT.

**Table 2:** Descriptive summary of HR, QT interval, and QTc interval (N=50).^a^

		Values			*P* value
		Mean (SD)	Range	Median (IQR)	
**Platform**					
	HR^b^ (bpm)	77.17 (10.70)	53.50-97.50	76.5 (68.12-84.88)	N/A^c^
	QT interval (ms)	389.9 (33.95)	293.5-443.5	388.5 (369.9-417.9)	N/A
	QTc^d^ interval (ms)	434.4 (32.91)	305-487.5	437.5 (423.4-459.9)	N/A
**12-lead ECG^e^**					
	HR (bpm)	74.42 (10.44)	54-100	74 (66.5-80.75)	<.001
	QT interval (ms)	383.6 (26.63)	342-442	378 (362.5-406)	.005
	QTc interval (ms)	423.9 (23.16)	379-486	422.5 (408-438.8)	.001
**ECG lead I**					
	HR (bpm)	75.49 (12.17)	47-98	75 (67-84)	.047
	QT interval (ms)	368.9 (28.01)	318-429	365.5 (350.2-389.8)	<.001
	QTc interval (ms)	411.1 (27.30)	359-481	414 (392-426)	<.001
**ECG lead II**					
	HR (bpm)	74.98 (12.05)	47-105	75 (67-84)	.012
	QT interval (ms)	371.5 (31.35)	293-468	370 (354-390.8)	<.001
	QTc interval (ms)	412.4 (34.34)	336-494	417 (387-436)	<.001

^a^Statistical comparison between measurements from the platform and 12-lead ECG were done using Wilcoxon sign rank test.

^b^HR: heart rate.

^c^N/A: not available.

^d^QTc: corrected QT.

^e^ECG: electrocardiogram.

**Table 3:** Correlation between devices.

Platform measures with...	Intraclass correlation: consistency (95% CI)	Intraclass correlation: agreement (95% CI)	Pearson correlation (95% CI)
HR^a^ from 12-lead ECG^b^	0.88 (0.79-0.93)	0.85 (0.69-0.92)	0.87 (0.79-0.93)
HR (lead I)	0.75 (0.59-0.85)	0.74 (0.58-0.84)	0.75 (0.60-0.85)
HR (lead II)	0.79 (0.65-0.87)	0.77 (0.62-0.87)	0.79 (0.66-0.88)
QTc^c^ interval from 12-lead ECG	0.40 (0.14-0.61)	0.38 (0.13-0.59)	0.43 (0.17-0.63)
QTc interval (lead I)	0.42 (0.16-0.63)	0.33 (0.02-0.57)	0.43 (0.17-0.63)
QTc interval (lead II)	0.41 (0.15-0.62)	0.34 (0.05-0.57)	0.41 (0.14-0.62)
QT interval from 12-lead ECG	0.68 (0.49-0.80)	0.66 (0.48-0.80)	0.69 (0.52-0.82)
QT interval (lead I)	0.53 (0.30-0.70)	0.43 (0.09-0.66)	0.54 (0.31-0.71)
QT interval (lead II)	0.56 (0.34-0.73)	0.49 (0.17-0.70)	0.56 (0.34-0.73)

^a^HR: heart rate.

^b^ECG: electrocardiogram.

^c^QTc: corrected QT.

**Table 4:** Association of the platform’s QT/QTc intervals with the 12-lead electrocardiogram’s QT/QTc intervals.^a^

			12-lead									Lead I									Lead II						
			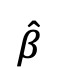 ^b,c^ (SE)		*P* value		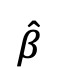 ^b,d^ (SE)		*P* value		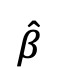 ^b,c^ (SE)			*P* value		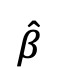 ^b,d^ (SE)		*P* value		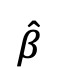 ^b,c^ (SE)			*P* value		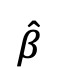 ^b,c^ (SE)		*P* value
**QTc^e,f^**			0.30 (0.09)		.002		0.31 (0.09)		.001		0.35 (0.11)			.002		0.30 (0.12)		.01		0.42 (0.14)			.002		0.30 (0.14)		.03
	Age					0.30 (0.22)		.18							0.33 (0.29)		.26							0.04 (0.36)		.92	
	Male					–10.66 (6.03)		.08							–10.84 (7.87)		.26							–22.29 (9.41)		.92	
	HR^g,h^					–1.93 (0.52)		<.001							–0.93 (0.43)		.03							–1.62 (0.56)		.006	
**QT^f^**			0.54 (0.08)		<.001		0.53 (0.08)		<.001		0.44 (0.10)			<.001		0.41 (0.09)		<.001		0.52 (0.11)			<.001		0.48 (0.12)		<.001
	Age					0.37 (0.24)		.13							0.47 (0.26)		.08							0.19 (0.33)		.58	
	Male					0.54 (6.02)		.93							2.03 (6.47)		.75							–8.13 (8.11)		.32	
	HR					–0.29 (0.53)		.59							1.09 (0.38)		.006							0.54 (0.53)		.31	

^a^The QT/QTc intervals from the 12-lead electrocardiogram were modeled using multiple linear regression with QT/QTc intervals from the platform as the main predictor, adjusted for age, sex, and change in HR.

^b^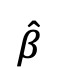: parameter estimates.

^c^simple linear regression.

^d^multiple linear regression.

^e^QTc: corrected QT.

^f^QTc/QT measurements were from the platform.

^g^HR: heart rate.

^h^Change in HR between the platform and electrocardiogram.

**Figure 1:**
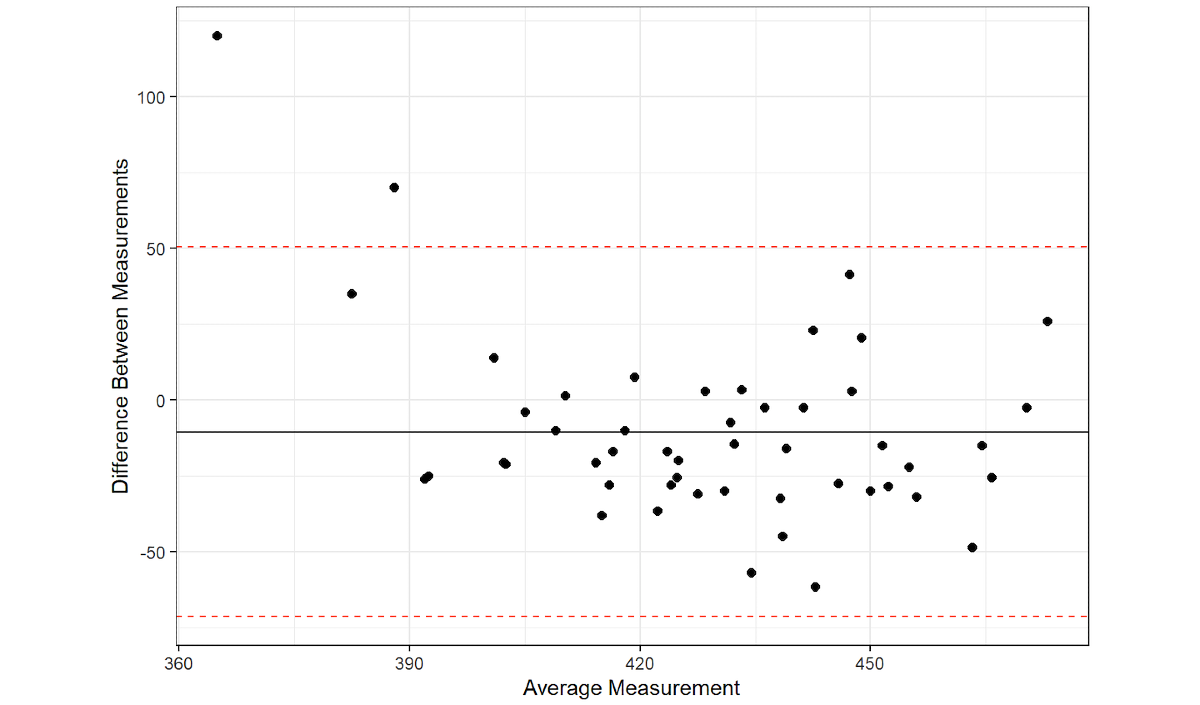
The 12-lead corrected QT (QTC) with AccurBeat QTc.

**Figure 2:**
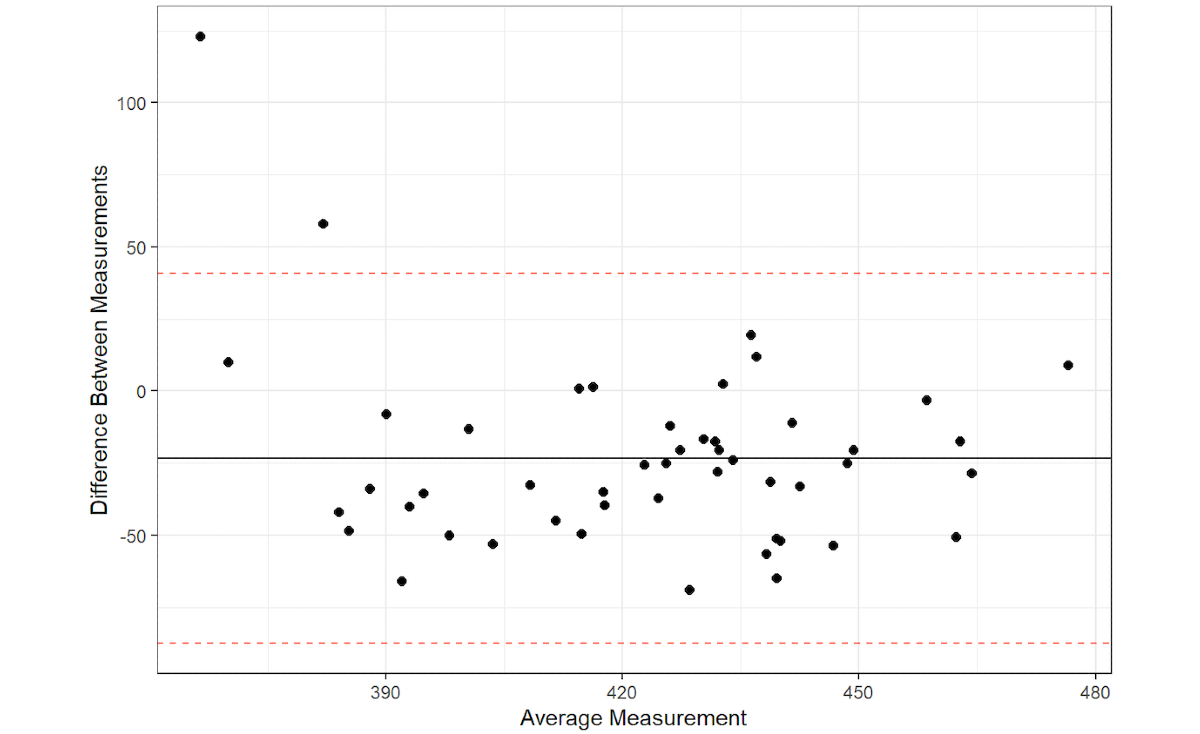
Lead I corrected QT (QTc) with AccurBeat QTc.

**Figure 3:**
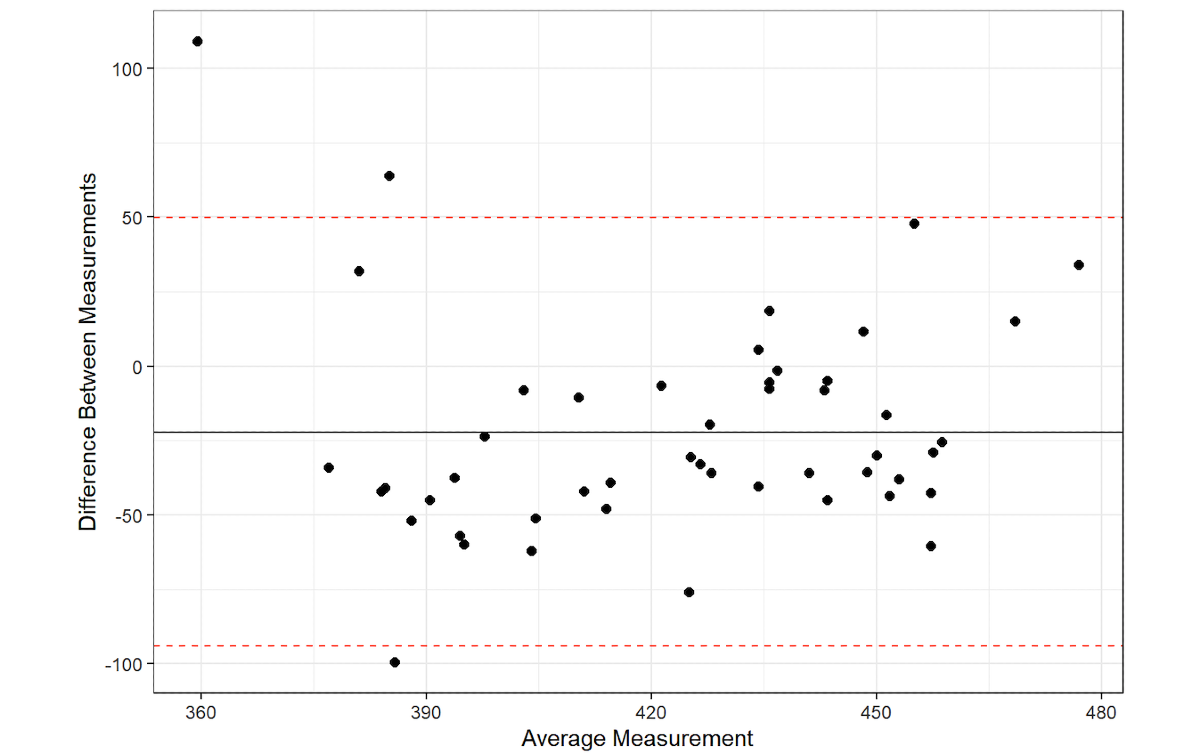
Lead II corrected QT (QTc) with AccurBeat QTc.

**Figure 4:**
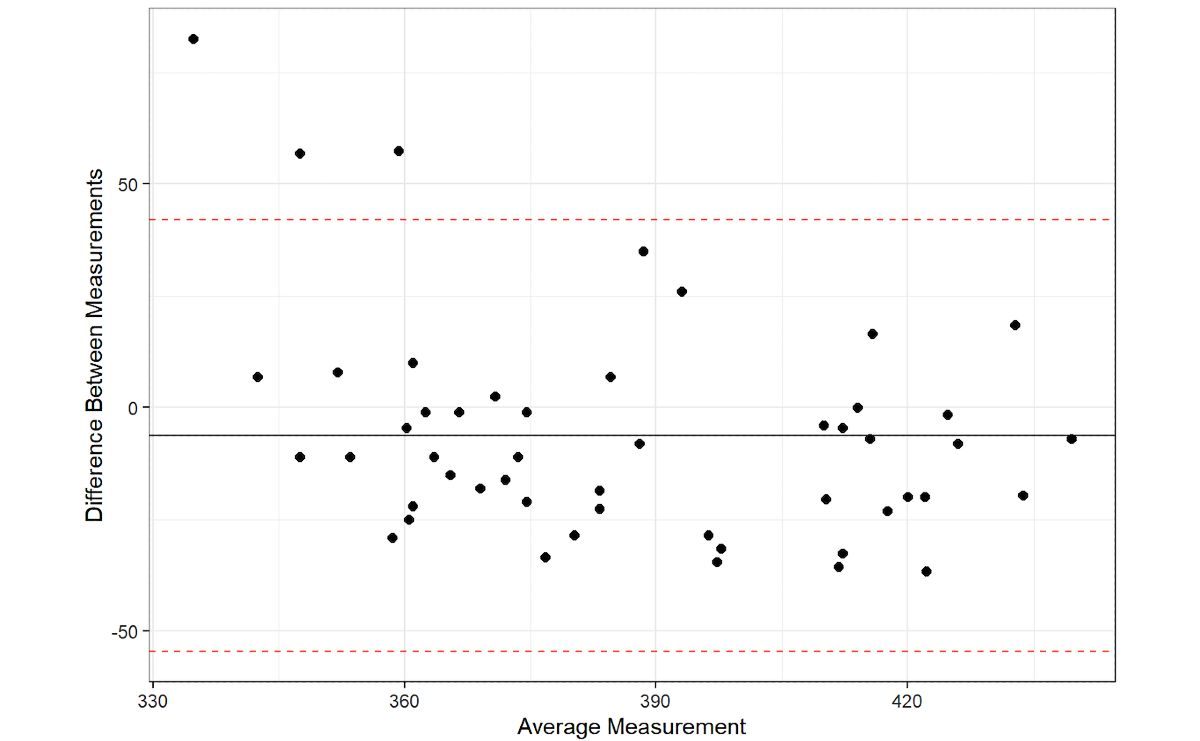
The 12-lead QT with AccurBeat QT.

**Figure 5:**
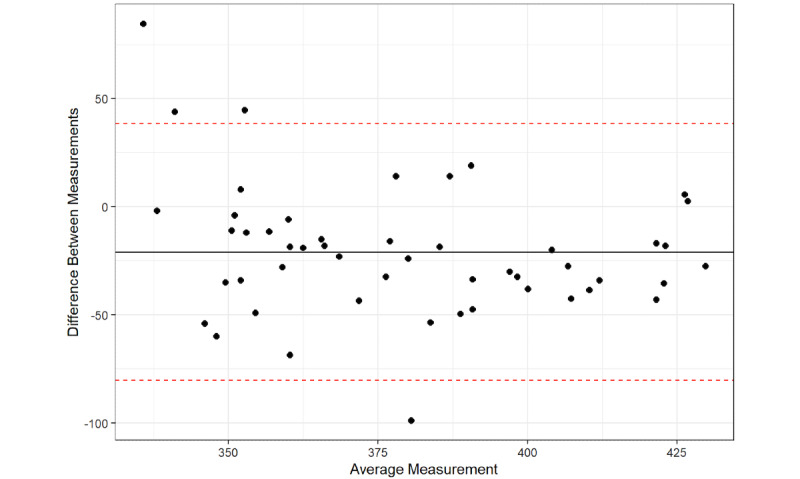
Lead I QT with AccurBeat QT.

**Figure 6:**
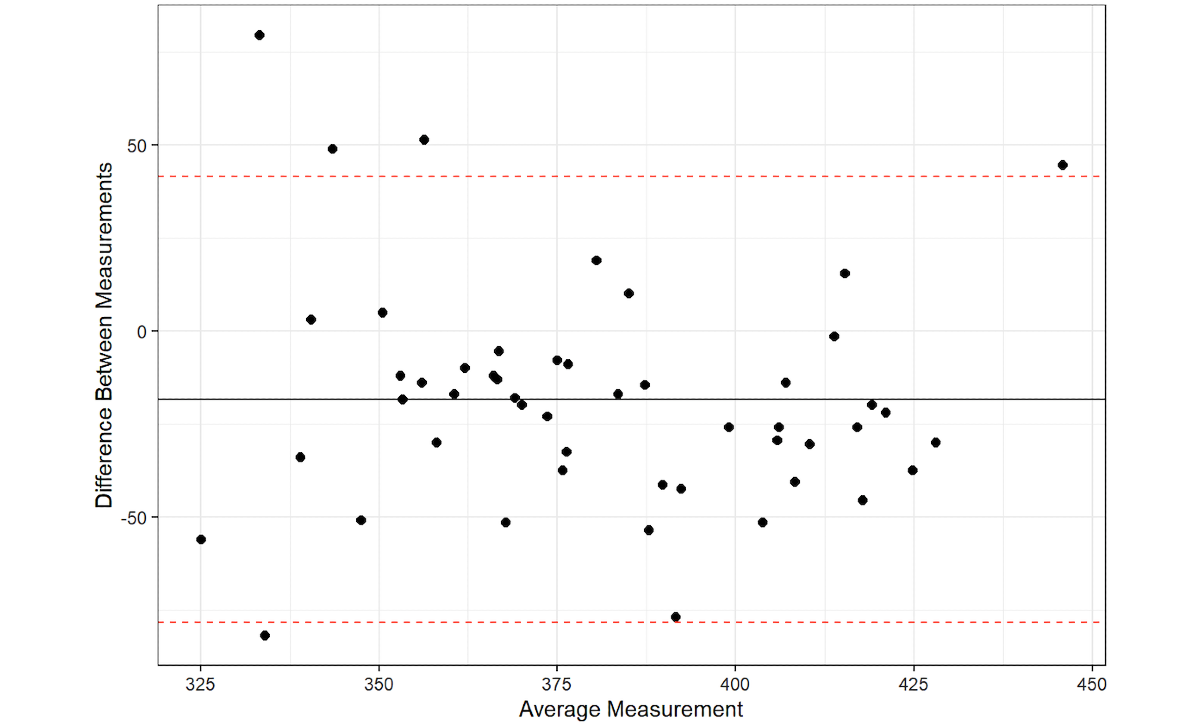
Lead II QT with AccurBeat QT.

The Bland-Altman plot results found that 96% of the average QTc interval measurements between the platform and the QTc intervals from the 12-lead ECG were within the 95% confidence limit of the average difference between the two measurements (Figures 1-6), with a mean difference of –10.5 (95% LoA –71.43, 50.43). The Bland-Altman analysis detected a significant proportional bias between the AccurBeat QTc interval and the QTc interval from the 12-lead ECG (*P*=.008). Over 95% of the average QTc interval measurements between the platform and QTc intervals from the 12-lead ECG (lead I) were within the 95% confidence limit of the average difference between the two measurements, with a mean difference of –23.45 (95% LoA –87.62, 40.72). The Bland-Altman analysis detected no significant proportional bias between the AccurBeat QTc intervals and the QTc intervals from the ECG lead I (*P*=.14). Over 93% of the average QTc interval measurements between the platform and the 12-lead ECG (lead II) were within the 95% confidence limit of the average difference between the two measurements, with a mean difference of –22.2 (95% LoA –94.15, 49.82). The Bland-Altman analysis detected no significant proportional bias between the AccurBeat QTc intervals and the QTc intervals from the ECG lead II (*P*=.81).

A total of 94% of the average QT interval measurements between the platform and QT intervals from the 12-lead ECG were within the 95% CI for the average difference between the two measurements, with a mean difference of –6.3 (95% LoA –54.54, 41.94). The Bland-Altman analysis detected a significant proportional bias between the AccurBeat QT intervals and the QT intervals from the 12-lead ECG (*P*=.02). A total of 94% of the average QT interval measurements between the platform and the QT intervals from the 12-lead ECG (lead I) were within the 95% CI for the average difference between the two measurements, with a mean difference of –21.08 (95% LoA –80.34, 38.18). The Bland-Altman analysis detected no significant proportional bias between the AccurBeat QT intervals and the QT intervals from the ECG lead I (*P*=.12). A total of 90% of the average QT interval measurements between the platform and the 12-lead ECG (lead II) were within the 95% confidence limit of the average difference between the two measurements, with a mean difference of –18.48 (95% LoA –78.44, 41.47). The Bland-Altman analysis detected no significant proportional bias between the AccurBeat QT interval and the QT from the ECG lead II (*P*=.51).

## Discussion

### Principal Findings

This study used the Apple smartwatch coupled with the platform to assess QT and QTc intervals, and showed reasonable accuracy with measures derived from conventional 12-lead ECG tracing in healthy controls without known cardiovascular disease. While associations ranged from low to moderate-high for the various measures of comparison, more than 90% of the average QT interval measurements between the platform and QT intervals from the 12-lead ECG were within the 95% CI of the average difference between the two measurements. Additionally, the technology platform posed no bias in terms of under- or overestimation. Moreover, intraclass measures of consistency and agreement as well as Pearson correlations were all higher for the QT interval than for the QTc interval.

### Comparison to Prior Work

The measurement of QT intervals is an important consideration in the identification of individuals at increased risk for ventricular tachycardia and sudden cardiac death [8-10]. QT interval monitoring is also important in terms of monitoring patients initiated and dose titrated on various classes of medications. The QT interval represents the time interval from onset of ventricular depolarization to the end of depolarization, is measured from the start of the q wave to the end of the T wave, and is usually obtained from a 12-lead ECG [8]. While computerized automatically derived values are often used for clinical purposes, some authors have advocated that manual measurement is more accurate [24]. However, manual QT interval assessment is tedious and time-consuming with greater interobserver variability, and physicians often select one complex from one lead (lead II) and one to measure [25]. Both techniques are limited by difficult T wave morphologies and the presence of u waves [26].

In recent years, there has been growing interest in the utility of wearables and smartphones [27] for remote ECG monitoring, which has been largely accelerated by the COVID-19 pandemic. In this regard, the Apple Watch has been shown to be a useful screening strategy for the detection of atrial fibrillation [28]. The measurement of QT intervals using smartwatch technology represents an important extension of remote monitoring and poses advantages regarding cost and convenience, and the ability of prolonged monitoring. This study showed comparable values from the Apple smartwatch coupled with the platform technology to those from a 12-lead ECG, from lead I alone and from lead II.

In the multivariate analysis, age did not impact the predictive value of AccurKardia’s technology even though age-related changes in QT interval have been previously reported [29]. Although there was strong agreement and consistency for HR values, the comparisons were less strong for QTc intervals than for QT intervals. These findings are not unexpected as QTc interval comparisons include potential measurement errors from two values (QT interval and cycle length), and the QTc interval was calculated by Bazett’s [20] formula, in which small differences in HR translate into relatively large differences in QT interval correction. Additionally, while consistency and agreement were similar for most comparisons, agreement was lower in comparisons of smartwatch-obtained values with leads I and II. This finding was expected as QT intervals were measured from 2 to 4 beats in these leads.

Important differences in acquisition methods that could also contribute to potential sources of error include the fact that the platform’s method includes a total of 60 seconds of recording, whereas a 12-lead ECG is recorded over 10 seconds. Since these participants were relatively young (mean age 46 years) and healthy, respiratory variations in HR due to sinus arrhythmia over the short-term recording of a 12-lead ECG could adversely affect comparisons [30]. It may be that 60 seconds of focused application of the Apple Watch was subject to less sinus arrhythmia than 10 seconds of a resting ECG [31]. It remains unclear as to whether comparisons would be even stronger if a longer 12-lead recording time were to be performed. Additionally, the inclusion of patients with heart disease would likely reduce beats due to variations in HR and would expand the range of QT intervals to include prolonged values; in this regard, the design of the study was a conservative one. Typically, there is variability in values obtained from the 12 different leads of an ECG, a phenomenon known as QT dispersion [32,33]. Therefore, the algorithm measured the QT interval from one specific lead as an approximation of the longest QT interval of the 12 leads.

### Limitations

There are a number of limitations in this study that warrant mention. First, smartwatch and 12-lead ECG recordings were obtained sequentially and not simultaneously. However, the brief intervening period should not be expected to substantially impact results. Second, this pilot study evaluated healthy individuals without known heart disease, and the results cannot be extrapolated to those with known heart disease. The research group intends to perform a follow-up study including patients with heart disease. Third, the 12-lead ECG measurements were not made by multiple people but by a single experienced investigator (JL). In future studies, the study group plans to get the 12-lead ECG measured by more than one investigator to take into account interobserver variability.

### Strengths

A strength of the study is that it included a racially diverse group of study participants in which 50% were African American. While darker skin color has been reported to adversely affect smartwatch recording capabilities [34], we did not perform racial comparisons.

### Conclusion

Despite these limitations, we conclude that QT and QTc intervals obtained by the Apple smartwatch coupled with the platform are comparable to those from a 12-lead ECG. Future research is planned to build upon the learnings in this study, expanding to a larger sample of patients that includes patients with cardiac disease as well as multiple cardiologists and assessments of their reliability with regard to manual annotation of ECG tracings. With these learnings and further refinements, remote monitoring using this technology holds promise for the identification of QT prolongation.

## Abbreviations

AI: artificial intelligence
ECG: electrocardiogram
HR: heart rate
LoA: limits of agreement
QTc: corrected QT
SUNY: State University of New York
VIF: variance inflation factor

## Data Availability

The data sets used or analyzed during this study will be available from the corresponding author upon reasonable request.

## References

[1] Virani SS, Alonso A, Benjamin EJ, Bittencourt MS, Callaway CW, Carson AP, Chamberlain AM, Chang AR, Cheng S, Delling FN, Djousse L, Elkind MS, Ferguson JF, Fornage M, Khan SS, Kissela BM, Knutson KL, Kwan TW, Lackland DT, Lewis TT, Lichtman JH, Longenecker CT, Loop MS, Lutsey PL, Martin SS, Matsushita K, Moran AE, Mussolino ME, Perak AM, Rosamond WD, Roth GA, Sampson UK, Satou GM, Schroeder EB, Shah SH, Shay CM, Spartano NL, Stokes A, Tirschwell DL, VanWagner LB, Tsao CW, American Heart Association Council on Epidemiology and Prevention Statistics Committee and Stroke Statistics Subcommittee (2020). Heart disease and stroke statistics-2020 update: a report from the American Heart Association. Circulation.

[2] Fryar CD, Chen T, Li X (2012). Prevalence of uncontrolled risk factors for cardiovascular disease: United States, 1999-2010. NCHS Data Brief.

[3] Mant J, Fitzmaurice DA, Hobbs FDR, Jowett S, Murray ET, Holder R, Davies M, Lip GYH (2007). Accuracy of diagnosing atrial fibrillation on electrocardiogram by primary care practitioners and interpretative diagnostic software: analysis of data from screening for atrial fibrillation in the elderly (SAFE) trial. BMJ.

[4] Ribeiro AH, Ribeiro MH, Paixão GMM, Oliveira DM, Gomes PR, Canazart JA, Ferreira MPS, Andersson CR, Macfarlane PW, Meira W, Schön TB, Ribeiro ALP (2020). Automatic diagnosis of the 12-lead ECG using a deep neural network. Nat Commun.

[5] Veronese G, Germini F, Ingrassia S, Cutuli O, Donati V, Bonacchini L, Marcucci M, Fabbri A, Italian Society of Emergency Medicine (SIMEU) (2016). Emergency physician accuracy in interpreting electrocardiograms with potential ST-segment elevation myocardial infarction: Is it enough?. Acute Card Care.

[6] Porta-Sánchez A, Gilbert C, Spears D, Amir E, Chan J, Nanthakumar K, Thavendiranathan P (2017). Incidence, diagnosis, and management of QT prolongation induced by cancer therapies: a systematic review. J Am Heart Assoc.

[7] Nassar Junior AP (2021). COVID-19 pandemic and the opportunity to accelerate remote monitoring of patients. J Bras Pneumol.

[8] Postema P, Wilde A (2014). The measurement of the QT interval. Curr Cardiol Rev.

[9] Lepeschkin E, Surawicz B (1952). The measurement of the Q-T interval of the electrocardiogram. Circulation.

[10] Giudicessi JR, Schram M, Bos JM, Galloway CD, Shreibati JB, Johnson PW, Carter RE, Disrud LW, Kleiman R, Attia ZI, Noseworthy PA, Friedman PA, Albert DE, Ackerman MJ (2021). Artificial intelligence-enabled assessment of the heart rate corrected QT interval using a mobile electrocardiogram device. Circulation.

[11] Curran M, Splawski I, Timothy K, Vincent GM, Green E, Keating M (1995). A molecular basis for cardiac arrhythmia: HERG mutations cause long QT syndrome. Cell.

[12] Wang Q, Shen J, Splawski I, Atkinson D, Li Z, Robinson JL, Moss AJ, Towbin JA, Keating MT (1995). SCN5A mutations associated with an inherited cardiac arrhythmia, long QT syndrome. Cell.

[13] Gaita F, Giustetto C, Bianchi F, Wolpert C, Schimpf R, Riccardi R, Grossi S, Richiardi E, Borggrefe M (2003). Short QT syndrome. Circulation.

[14] Duncker D, Ding WY, Etheridge S, Noseworthy PA, Veltmann C, Yao X, Bunch TJ, Gupta D (2021). Smart wearables for cardiac monitoring-real-world use beyond atrial fibrillation. Sensors (Basel).

[15] Dinh-Le C, Chuang R, Chokshi S, Mann D (2019). Wearable health technology and electronic health record integration: scoping review and future directions. JMIR Mhealth Uhealth.

[16] Hannun AY, Rajpurkar P, Haghpanahi M, Tison GH, Bourn C, Turakhia MP, Ng AY (2019). Cardiologist-level arrhythmia detection and classification in ambulatory electrocardiograms using a deep neural network. Nat Med.

[17] Strik M, Caillol T, Ramirez FD, Abu-Alrub S, Marchand H, Welte N, Ritter P, Haïssaguerre M, Ploux S, Bordachar P (2020). Validating QT-interval measurement using the Apple Watch ECG to enable remote monitoring during the COVID-19 pandemic. Circulation.

[18] Maille B, Wilkin M, Million M, Rességuier N, Franceschi F, Koutbi-Franceschi L, Hourdain J, Martinez E, Zabern M, Gardella C, Tissot-Dupont H, Singh JP, Deharo J, Fiorina L (2021). Smartwatch electrocardiogram and artificial intelligence for assessing cardiac-rhythm safety of drug therapy in the COVID-19 pandemic. The QT-logs study. Int J Cardiol.

[19] Spaccarotella CAM, Migliarino S, Mongiardo A, Sabatino J, Santarpia G, De Rosa S, Curcio A, Indolfi C (2021). Measurement of the QT interval using the Apple Watch. Sci Rep.

[20] Bazett HC (1997). An analysis of the time-relations of electrocardiograms. Ann Noninvasive Electrocardiol.

[21] Bland JM, Altman DG (1986). Statistical methods for assessing agreement between two methods of clinical measurement. Lancet.

[22] Giavarina D (2015). Understanding Bland Altman analysis. Biochem Med (Zagreb).

[23] Shrout PE, Fleiss JL (1979). Intraclass correlations: uses in assessing rater reliability. Psychol Bull.

[24] Indraratna P, Tardo D, Delves M, Szirt R, Ng B (2020). Measurement and management of QT interval prolongation for general physicians. J Gen Intern Med.

[25] Panicker GK, Karnad DR, Natekar M, Kothari S, Narula D, Lokhandwala Y (2009). Intra- and interreader variability in QT interval measurement by tangent and threshold methods in a central electrocardiogram laboratory. J Electrocardiol.

[26] Tran H, Fan C, Tu W, Kertland H, Li L, Kluger J, Chow MS (1998). QT measurement: a comparison of three simple methods. Ann Noninv Electrocard.

[27] Beers L, van Adrichem LP, Himmelreich J, Karregat EPM, de Jong JSSG, Postema PG, de Groot JR, Lucassen WAM, Harskamp RE (2021). Manual QT interval measurement with a smartphone-operated single-lead ECG versus 12-lead ECG: a within-patient diagnostic validation study in primary care. BMJ Open.

[28] Perez MV, Mahaffey KW, Hedlin H, Rumsfeld JS, Garcia A, Ferris T, Balasubramanian V, Russo AM, Rajmane A, Cheung L, Hung G, Lee J, Kowey P, Talati N, Nag D, Gummidipundi SE, Beatty A, Hills MT, Desai S, Granger CB, Desai M, Turakhia MP, Apple Heart Study Investigators (2019). Large-scale assessment of a Smartwatch to identify atrial fibrillation. N Engl J Med.

[29] Vink AS, Clur SB, Wilde AA, Blom NA (2018). Effect of age and gender on the QTc-interval in healthy individuals and patients with long-QT syndrome. Trends Cardiovasc Med.

[30] Soos MP, McComb D (2022). Sinus Arrhythmia.

[31] Hatfield BD, Spalding TW, Santa Maria DL, Porges SW, Potts JT, Byrne EA, Brody EB, Mahon AD (1998). Respiratory sinus arrhythmia during exercise in aerobically trained and untrained men. Med Sci Sports Exerc.

[32] Hnatkova K, Malik M (2020). Sources of QTc variability: implications for effective ECG monitoring in clinical practice. Ann Noninvasive Electrocardiol.

[33] Statters D, Malik M, Ward D, Camm A (1994). QT dispersion: problems of methodology and clinical significance. J Cardiovasc Electrophysiol.

[34] Ray I, Liaqat D, Gabel M, de Lara E (2021). Skin tone, confidence, and data quality of heart rate sensing in WearOS smartwatches. IEEE International Conference on Pervasive Computing and Communications Workshops and other Affiliated Events.

